# Hemorrhagic pancreatic schwannoma mimicking a pancreatic neuroendocrine tumor

**DOI:** 10.1093/omcr/omag189

**Published:** 2026-10-05

**Authors:** Ryoma Sakamoto, Ken Hayashi, Shion Ando, Yuto Kawate, Akinari Miyazaki, Hiroshi Kusanagi

**Affiliations:** Department of Gastrointestinal Surgery, Kameda Medical Center, 929, Higashi-cho, Kamogawa, Chiba 2960041, Japan; Department of Gastrointestinal Surgery, Kameda Medical Center, 929, Higashi-cho, Kamogawa, Chiba 2960041, Japan; Department of Pathology, Kameda Medical Center, 929, Higashi-cho, Kamogawa, Chiba 2960041, Japan; Department of Gastrointestinal Surgery, Kameda Medical Center, 929, Higashi-cho, Kamogawa, Chiba 2960041, Japan; Department of Gastrointestinal Surgery, Kameda Medical Center, 929, Higashi-cho, Kamogawa, Chiba 2960041, Japan; Department of Gastrointestinal Surgery, Kameda Medical Center, 929, Higashi-cho, Kamogawa, Chiba 2960041, Japan

A 67-year-old woman underwent computed tomography (CT) for evaluation of an abnormal lung opacity, which incidentally revealed a pancreatic mass. Contrast-enhanced CT revealed a well-circumscribed 31 × 23 mm pancreatic mass with heterogeneous hypoattenuating areas and progressive peripheral enhancement (Fig. 1a). The differential diagnoses included pancreatic neuroendocrine tumor (pNET), pancreatic schwannoma (PS), and paraganglioma. ^123^I-metaiodobenzylguanidine scintigraphy revealed only faint uptake; a functional paraganglioma was considered unlikely after an endocrinology consultation. The negative results of somatostatin receptor scintigraphy indicated a lower likelihood of pNET. Based on the well-circumscribed appearance and favorable location of the mass, laparoscopic tumor enucleation was performed. The patient was discharged 7 days after surgery without complications. Histological examination revealed a well-encapsulated tumor with extensive internal hemorrhaging (Fig. 1b). Histologically, the tumor consisted of wavy spindle cells that formed alternating Antoni A and B areas without significant atypia or mitotic activity. The tumor cells were positive for S100 and negative for synaptophysin, chromogranin A, and INSM1, which confirmed the diagnosis of PS. The pancreatic acinar tissue was identified adjacent to the tumor capsule, which supported a pancreatic origin. The resection margins were negative. PS is an extremely rare neoplasm that accounts for approximately 0.03% of all pancreatic tumors [1]. PS can undergo hemorrhagic or cystic degeneration [2], whereas pNET contains necrotic components [3]. Extensive intra-tumoral hemorrhage produced a heterogeneous appearance, which mimics a pNET with internal necrosis. The preoperative diagnosis of PS is challenging because it frequently mimics other pancreatic neoplasms. Zhang et al. reported that 29% of the patients with PS were preoperatively diagnosed with pNET [4]. This case illustrates a clear radiologic–pathologic correlation in a pancreatic schwannoma, wherein extensive intratumoral hemorrhage produced a heterogeneous appearance that mimicked pNET with internal necrosis.

**Figure 1:**
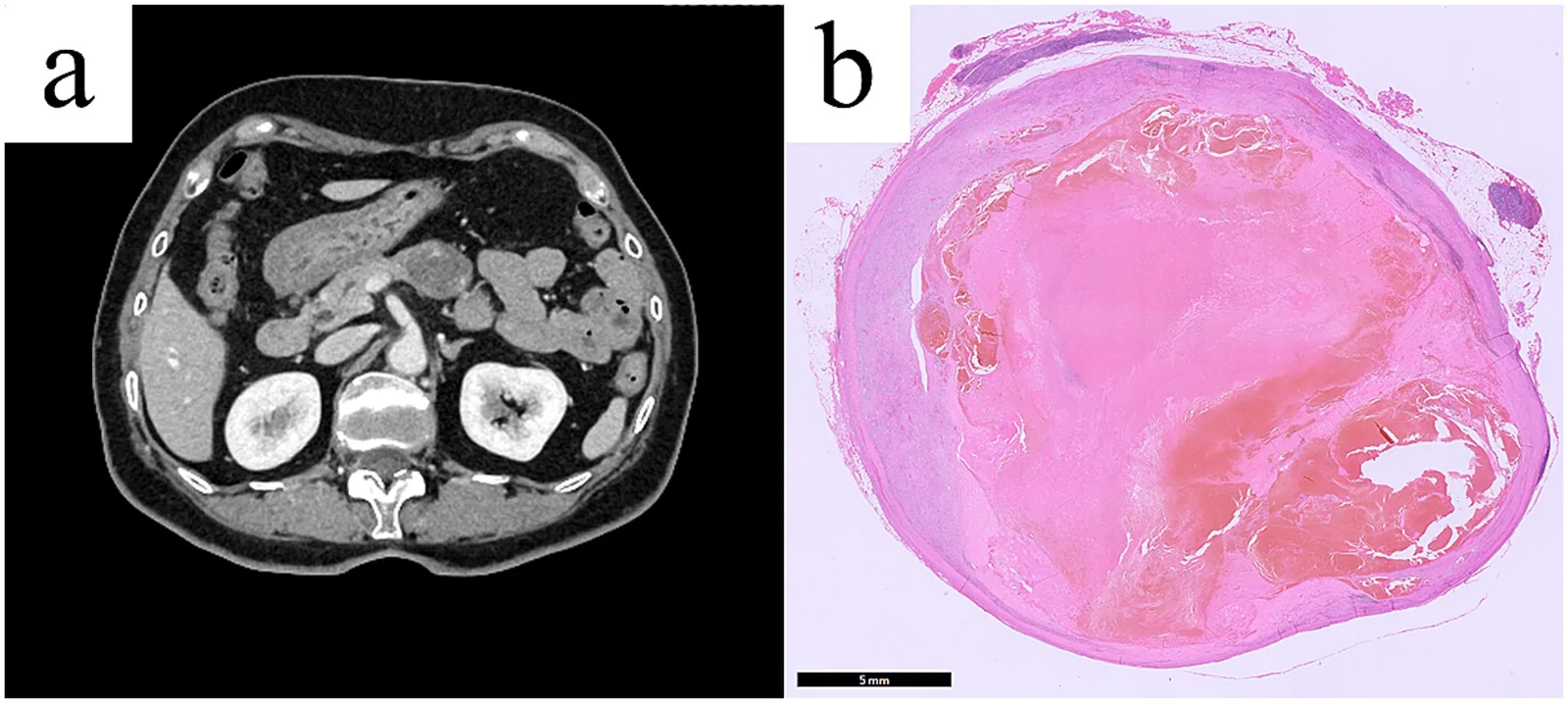
(a) Computed tomography revealed a well-circumscribed mass with internal heterogeneous hypoattenuation. Small intratumoral foci show enhancement similar to that of adjacent blood vessels. (b) Low-power view (hematoxylin and eosin stain). The tumor is well circumscribed and shows extensive internal hemorrhage and degeneration.
